# The Journal Impact Factor is under attack – use the CAPCI factor instead

**DOI:** 10.1186/s12916-016-0773-5

**Published:** 2017-01-16

**Authors:** Eleftherios P. Diamandis

**Affiliations:** 1 Department of Pathology and Laboratory Medicine, Mount Sinai Hospital, Toronto, ON Canada; 2 Department of Clinical Biochemistry, University Health Network, Toronto, ON Canada

**Keywords:** Journal impact factor, CAPCI factor, Scientific impact, Journal quality

## Abstract

The uses and misuses of the Journal Impact Factor (JIF) have been thoroughly discussed in the literature. A few years ago, I predicted that JIF would soon be replaced, while another colleague argued the opposite. Over the past few months, attacks on JIF have intensified, with some publishing organizations gradually removing the indicator from their journals’ websites. Here, I argue that most, if not all of the misuses of JIF are related to its name. The word “impact” should be removed, since it implies an influential attribute, either for the journals, their published papers, or their authors. I propose instead the use of a new name, the “CAPCI factor”, standing for Citation Average Per Citable Item, which accurately describes what is represented by this measure.

## Editorial

More than 50 years ago, information scientist Eugene Garfield and colleagues described a simple publication indicator, known as the Journal Impact Factor (JIF) [1]. The JIF indicates the average number of citations received by papers published in a specific journal over a specific period of time. The calculation is very elementary math; divide the number of citations of all papers published in a journal over a specific time period (say 1, 2, or 5 years) by the number of papers published and …. Eureka! You have your number.

Like every other simple “discovery”, Garfield did not anticipate that this calculation was destined to become his greatest hit. This story is reminiscent of numerous other unlikely “successes”, one example being the polymerase chain reaction – a simple molecular biology technique that went on to make billions of dollars in commercial products and won a Nobel prize. Yet, why has the JIF enjoyed so much publicity in recent years? I believe that the JIF was born at just the right time, when explosive growth and revolutionary changes took place in academic publishing, mainly related to the electronic and digital era. Authors, publishers, funders, and other stakeholders were hungry for a metric that could separate, with a glimpse of eye, published gold from published mediocrity. But, is the JIF really telling you this?

Now, please allow me to divert, to make an analogy which has many similarities with the issue at hand. Many know that my favorite sport is tennis. Let us ask the question as to how many tennis balls, on average, a player is consuming over a certain period, say a year or two. Like the JIF, you can get your answer by dividing the number of sold balls by the number of the customers of a tennis shop. This is the Tennis Ball Impact Factor! Let us now ask if one shop should be considered ‘better’ than another if it sells more tennis balls per customer. It could indeed be that the better selling shop has better service, but other explanations may be at play, such as better advertising or a better location! Thus, selling more balls per customer (thus having higher Tennis Ball Impact Factor) may or may not be related to the quality of the shop. Let us now examine some other issues, not so much related to the shop but to its customers. Could a customer who buys balls from the best-selling shop claim that they could play better tennis than somebody who buys balls from a less popular shop? If the answer is yes (but unfortunately it isn’t), then I would rush to buy balls from that shop, in hopes of improving my game! Even better, I would buy balls from Roger Federer’s favorite shop, in hopes that my chances to win Wimbledon will skyrocket! I wish it were that simple!

With these thoughts in mind, I came to the conclusion, in 2009, that the JIF will not endure the test of time and would soon be replaced [2]. I predicted that Garfield’s simple calculation was not likely to become, or remain, the widely sought top quality indicator in scientific publishing, like Wimbledon is for tennis, the Master’s for golf, or the Kentucky Derby for horse racing. My colleague, Dr. E.J. Favaloro, had the opposite opinion. Our debate on this issue has been published [2, 3].

Of course, I am not the first to criticize the JIF. Numerous editors, authors, and Associations of Editors expressed concerns with the use and misuse of JIF, and these concerns will not be repeated here [4–6]. I briefly summarize that the consensus is that the JIF should not be used as a surrogate measure of quality of individual research papers or for assessing an author’s scientific contribution, nor used to hire, promote, or fund individuals [7].

The JIF debate is closely monitored by authors, editors, and publishers. Some leading journals, including *Nature* and *Science*, are trying to devise improved indicators of journal performance [8, 9]. For example, it is well-known that the distributions of citations among published papers are highly skewed to the right (see data in [9]) and the use of mean citation values leads to the estimation of a much higher JIF than by using the medians instead. For example, *Nature*’s JIF went from 38 (with means) to 24 (with medians). It is rather appalling that this simple, and seemingly better, calculation has not already been widely implemented. After all, we teach our undergraduates never to use parametric tests (including means) if the data distributions are not normal! Yet, the reason may be obvious – I would not imagine any editor or publishing executive who would adopt an improved measure of the JIF if it implies that their current JIF would go down! The reverse is likely also true, wherein nearly all editors would immediately adopt a revision of the current calculation if it were to improve their JIF. *Nature*, and some other elite journals, are a special case. Since they rein in scientific publishing prestige, the JIF is of no consequence to them. Authors would still prefer them from any other journal with a much higher JIF.

## Change the name of the JIF

Herein, I present an amusing suggestion to modify the JIF calculation in a way which, I suspect, would be highly popular. Let us calculate the JIF as usual, but include a per capita correction (i.e., per million) for the country that the journal is published in. Assume the current JIF of a journal published in the USA to be 20. Correcting per million, the new JIF would be 20/360 = 0.06. However, if published in Monaco (0.04 million inhabitants), the JIF would be 20/0.04 = 500. Based on this, I would predict that most, if not all, journals published in the USA will move their editorial offices to Monaco within 1 year, even if the newly elected US President would likely oppose it!

The voices against JIF are now becoming louder [7–10]. The American Society for Microbiology has already announced plans to remove the JIF from their journal’s website as well as from their marketing and advertising efforts. In 2013, I predicted that the role of journals will change, even more, to become repositories of information to be continuously assessed by readers [5]. If this happens, the JIF will become an irrelevant measure.

In closing, I believe that many of the problems associated with JIF are related to its name. Surprisingly, this issue has not been frequently discussed in previous debates. The word “impact” implies that this number carries influential information of some sort, either for the journal (somewhat legitimate) or for the authors (totally illegitimate). I am thus proposing that the current name is replaced by the term CAPCI factor, or Citation Average Per Citable Item, which accurately depicts what the factor represents. While I have very frequently seen claims such as “my work has high impact since it is published in a high impact journal”, I highly doubt that any sensible scientist would claim that “my work has high impact because it is published in a journal with high CAPCI factor”.

So, where would you buy your tennis balls? I would advise finding a shop that sells more balls, not so much that this will help your game, or that the balls will be better, but because you will likely get a lower price!
